# An Efficient CNN for Hand X-Ray Classification of Rheumatoid Arthritis

**DOI:** 10.1155/2021/6712785

**Published:** 2021-06-14

**Authors:** Gitanjali S. Mate, Abdul K. Kureshi, Bhupesh Kumar Singh

**Affiliations:** ^1^Department of Electronics and Telecommunication, JSPM's Rajarshi Shahu College of Engineering, Pune 411033, India; ^2^Department of Electronics, Maulana Mukhtar Ahmad Nadvi Technical Campus, Malegaon 423203, India; ^3^Arba Minch Institute of Technology, Arba Minch University, Arba Minch, Ethiopia

## Abstract

Hand Radiography (RA) is one of the prime tests for checking the progress of rheumatoid joint inflammation in human bone joints. Recognizing the specific phase of RA is a difficult assignment, as human abilities regularly curb the techniques for it. Convolutional neural network (CNN) is the center for hand recognition for recognizing complex examples. The human cerebrum capacities work in a high-level way, so CNN has been planned depending on organic neural-related organizations in humans for imitating its unpredictable capacities. This article accordingly presents the convolutional neural network (CNN) which has the ability to naturally gain proficiency with the qualities and anticipate the class of hand radiographs from an expansive informational collection. The reproduction of the CNN halfway layers, which depict the elements of the organization, is likewise appeared. For arrangement of the model, a dataset of 290 radiography images is utilized. The result indicates that hand X-rays are rated with an accuracy of 94.46% by the proposed methodology. Our experiments show that the network sensitivity is observed to be 0.95 and the specificity is observed to be 0.82.

## 1. Introduction

Joint inflammation is a state of spasms and pain in a human where two or more bones join together. Arthritis results in excruciating pain, joint swellings, joint stiffness, and poor function in joints. Healthy joints move naturally with the help tissue called the articular cartilage which is slippery and smooth. The tissues absorb pressure and shock which is caused by motion and pressure due to extreme stress on the joints where bones meet. This extreme pressure causes destruction of the tissues in the cartilage further resulting in arthritis. The only and primary cause of arthritis is the degradation of cartilage in the joints. The arthritis is generally classified in two types: (1) osteoarthritis and (2) rheumatoid arthritis.

In osteoarthritis, cartilage and underlying bone degenerate which causes pain and stiffness. Generally osteoarthritis is more common in middle age people. On the other hand, rheumatoid joint pain is an incendiary infection that influences joints.

Rheumatoid joint pain (RA) is an immune system condition and is quite possibly the most limit types of provocative joint pain. Beforehand, bone misfortune from rheumatoid joint pain (RA) was considered irreversible. Be that as it may, the presence of biologic specialists stayed away from the movement of bone misfortune related to RA [1]. The major imagery discoveries identified with RA advancement incorporate bone weakening, narrowing of joints, and periarticular osteoporosis, showing the annihilation of construction of the bones. Specifically, the event of bone disintegration proposes functioning RA; therefore the assessment of bone disintegration is exceptionally significant for the underlying analysis and later assessment of RA patients.

The procedures mentioned above are extremely tedious, in every case, since they require the assessment of all accessible joints and radiographs of the foot. Furthermore, it is additionally hard for radiologists and other related medical practitioners to recognize the changes in the reports from the accessible foot X-beams on the grounds that the progressions are frequently unpretentious. The utilization of a PC technique for appraisal of RA in a quantitative degree is consequently prudent in medical field.

In the past, a few methodologies were utilized like artificial neural network (ANN), support vector machine (SVM), and genetic algorithm (GA) strategies. In any case, these methodologies incorporate the computation of the adequacy highlights to order the injuries. Profound learning has as of late acquired revenue in the space of speculation.

By and large, the neural organization is classified as a convolution neural organization (CNN) or profound learning kind of neural organization. For visual imaging, the model of neural organization which has a profound learning as class is utilized generally.

For the developing a natural spoken-language processing system CNN provides the tools for development. Along with this CNN can be used in various areas like medical image analysis, audio and speech recognition, and computer vision.

## 2. Motivation

Around the globe, it is estimated that there are approximately 35–70 million individuals suffering from RA. Since there is no demonstrated solution for RA accessible yet, current medicines chiefly center around relief from excruciating pain, discomfort, irritation decrease, and easing back down or halting joint harm. To forestall irreversible joint harm, early location of RA is fundamental. For a successful clinical treatment, it is significant that the infection can be checked intently. Joint harm appraisal close by radiographs is a much-of-the-time utilized technique for observing the movement of RA.

The accompanying inquiries concerning RA have not yet been answered which enable us to further explore RA:

- What causes RA? How does it begin?
- Why does RA disturb the functioning of the joints?
- Does RA come to an end in a human? If not, why?

The inspiration driving such research is that the past related works of imagery investigation frameworks are not automized for the examination of RA as per factual properties like age factor and nonstop movement of RA action. There is no mechanized framework for perceiving the seriousness of RA stage by seeing the image of influenced territory like hand and knee. Such frameworks utilize a mix of image handling methods to deal with images and afterward measure the distance between the tibial and femoral bones as an authenticator of diagnosing the sickness. Along these lines, there is a requirement for a canny rheumatoid joint inflammation framework that invigorates the human visual investigation that ordinarily analyzes the rheumatoid joint pain contingent upon certain highlights like the limited distance between the tibial and femoral bones and furthermore the bone prods. Rheumatoid joint inflammation happens because of uncontrolled joint irritation, yet it is not right now known how and why this aggravation is set off.

Aside from this the third-stage patient does not recuperate. Due to long period of treatment towards RA, inflammation in bones is created. The intricacy increases with respect to age factor and sometimes to x beam. An entire day is required for getting the treatment. So we ought to have some automized framework which will be useful specially towards conclusion and movement of RA.

## 3. Related Work

The existing RA calculations from essential consideration records depend on a manual determination of the most important codes [2], which is to some degree emotional. ID of patients from the essential consideration information base enduring with RA with precision and unwavering quality is an intriguing errand. The greater part of the connected RA seriousness evaluating examines centers around recognizing finger joints or distinguishing bone disintegration on the X-beam image dataset rather than ultrasound images.

As of now, specialized progressions have prompted a significant improvement in image handling. Regardless of specialized progressions and the accessibility of various devices and tests, like ultrasound, magnetic resonance imaging (MRI), and conventional radiography (CR), CR stays the essential imaging device for RA.

While investigating multiple joints by MRI, the procedure takes an excessive lot of time and costs a lot consistently. Ultrasound is a client subordinate method of imaging that can measure emanation and synovial changes. An aggravation might be utilized to play out a little, decay and bone rarefaction joint evaluation that is an extremely agonizing and consistently deteriorating flare-up. It might likewise cause various imperfections and utilitarian issues in the bones. Such factors are in this manner generally imperative to identify in the beginning phases. All drugs, treatments, and procedures that have been undergone so far point just to lessening the manifestations and drawing out the movement of the infection. We can understand that the cases of RA are unsure and cures are not yet found in these painful cases.

To understand and further study RA, the authors in [3, 4] propose using computerized joint location and evaluation of hand radiographs. The prime objective of this method is location of joint position and joint edge. Besides, it is clarified by the edge time frame. The experiments were executed on five hand radiographical images of 16-bit grayscale with 2500 × 2000 pixels (0.1 × 0.1 mm). For this experiment and with manual joint outline, all the joints are comprehensively estimated.

In [4], the author has recommended a methodology using refreshed level set for programmed identification of the bone limit close by radiographs. The said strategy promises great extraction effectiveness. Because the pixel powers of bone joints and different pixels are closer in certain areas, the method fails to function as expected with respect to the images. Numerous conventional strategy studies like Volumetric Ultrasound and Infrared Imaging have detailed great execution in RA identification. Profound learning is becoming normal in the imaging field.

In [5, 6], joint space narrowing estimation has been very much contemplated and accomplished a superb evaluation in RA development. The MSGVF division calculation which is predominantly utilized for disintegration of bones and different CNN structures in [7–9], was assumed a basic part as it presents a recognizable proof and assessment of RA. The production of the different CNN models to identify RA has been discussed in [8–10] which quickly gives us a new beginning for the examination of RA. Experimenters have achieved great level in research using CNN for image processing with the aid of the learning networks, which adds a convolution and pooling design to a separate image database. In the diagnosis of RA, changing disease modifying antirheumatic drugs (DMARDs) and organic medications are utilized in [10–12].

As indicated by the writing, around 30–40% of RA patients [13] are not getting profited by exemplary first-line treatment which is MTX [14]. In 2019, a few significant logical advances were made in the clinical use of ML in rheumatology [15–20].

CNN is utilized mostly in imaging undertakings [21]. The authors of [22] report the successful utilization of CNN to advance computerized scoring of the activity of joint inflammation infection on ultrasound images. The authors of [23] utilize X-beams of RA patients to prepare and foster a profound CNN for joint obliteration appraisal. For quick therapy, early finding of patients with RA in danger of creating ongoing agony is clinically fitting. Lotsch et al. recommended that Machine Learning could be utilized to get RA useful boundaries from huge vault information, for instance, Disease Activity Score-28, and that these boundaries could exhibit the creation and level of constant agony [24].

A current experimental calculation with an exactness of 94% [25] was outflanked by a calculation prepared on innocent outcomes. The troupe Machine Learning strategies have shown guarantee to date in the dependable ID of RA and the savvy and precise evaluation of the illness [26, 27].

Sumitra Nair et al. [28] have proposed application depending on AI for investigation of EMG images designs for RA. Kernal LSK strategy is utilized for investigation of the appendage relocation in space [29].

In past examinations, numerous techniques for distinguishing injuries utilizing classifiers like a artificial neural network (ANN), support vector machine (SVM), and genetic algorithm (GA). As of late, profound learning has acquired revenue in the overall imaging field. In particular, numerous examinations have revealed a superior execution of the profound convolutional neural network (DCNN) compared to customary classifiers in image ID [30].

Cao and Mills et al. have proposed Towards Quantitative Assessment of RA utilizing Volumetric Ultrasound [31]. This calculation is used for sectioning (1) the 3D bone surface and (2) the 3D joint case district. They broaden 2D bone extraction strategies to 3D and make calculation more vigorous to the force misfortune because of surface ordinary's confronting away from occurrence acoustic shafts. The separated bone surfaces combined with a joint-explicit anatomical model are utilized to introduce a coarse restriction of the joint case locale. The joint case division is refined iteratively using a probabilistic spot model.

### 3.1. Image Analysis

The highest quality level for deciding underlying joint harm is radiography. Notwithstanding the wide utilization of radiography in joint assessment, PC supported indicative ultrasound frameworks have been created to arrange intra- and extra-articular pathologies with the likeness of joint space width appraisal [32] and joint aggravation assessment [33]. Additionally, the semiquantitative ultrasound evaluation of synovitis seriousness assists with deciding the infection action of RA presented in [20, 34]. RA assessment is presented as an issue of image arrangement [35, 36].

### 3.2. Data Augmentation

In this investigation, two RA ultrasound image datasets were utilized for preparing of the organization. One of the datasets incorporate 662 images of patients suffering from RA in MCP joints, and the other incorporates 315 images of PIP joint. The acquired examples were divided considering 80% data for training and the rest for test. A large number of ultrasound images do not prove to be sufficient for preparation of CNN and may lead to the major issue of overfitting [37]. Thus, for improving the capacity of organization speculation, information expansion was performed on preparing tests. In data increase, one can extend the preparation set by adjusting each class to protect the information name. The information expansion we took has the accompanying three strategies.

## 4. Preprocessing and Dataset Planning

For the development of this work, the images are collected from examined patients. Data entails 290 radiograph images of different sizes. The images in the dataset have been processed at earlier stage with removal of nose and images are resized to 100 × 100. The images are then appropriately cropped to retain similar aspect ratio which was present in the images of the original datasets. In Table 1, we see the description of the dataset and Table 2 shows us the spread of data. The dataset has been processed by resizing the images up to 100 × 100 and normalizing their values. The dataset was split into training dataset and testing dataset. The 75% images were used for training the model and 25% images are used for testing the model.

## 5. Methodology

### 5.1. System Architecture

**Figure 1:**
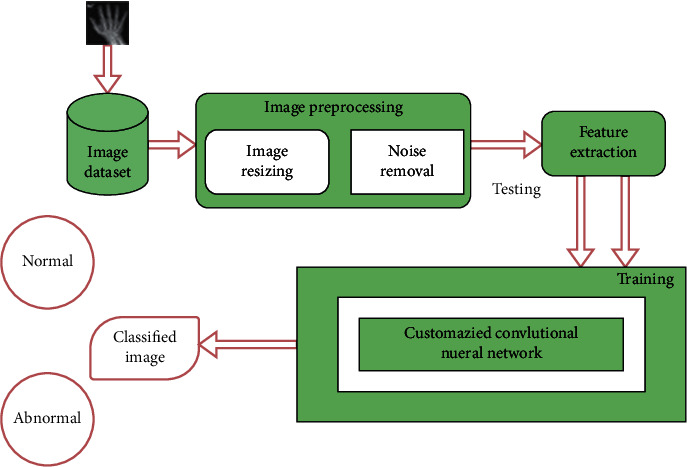
Layered network architecture for the detection of rheumatoid arthritis.

To identify RA cases with specified outcomes over the other popular strategies, the presented work alters the fundamental design of the regulated CNN organization. The presented CNN model does not restrict the quantity of layers that can be utilized; it enhances the number to fulfill the issue's interest. Moreover, middle convolution layers utilize different sizes of layers.  Figure 1 shows the proposed model's general gadget engineering. In the presented CNN the recently prepared image of size 28 × 28 expands the size of the layers alongside the initiating highlight. Mean pooling is utilized for reducing, by two stages, the image created from the convolutional layers. Further, the image is smoothed and taken care of by a method known as Multilayer Perception (MLP). Here, the image is processed further to a layer that is totally associated. At long last, the Sigmoid classifier gives us the likelihood of an image.

Standard neural organizations are adding to the issue of overfitting. Then again, CNN snatches advantage of the neighborhood qualities of the images; for example it manages input pixels which are situated close as well as far. In this experimentation, CNN was delivered to perceive different images dependent on the properties of the image.

The convolution network used in our experimentation is not only a profound neural organization with a few secret layers. It is a popular organization that copies how images are deciphered and recalled by the visuals. Consequently, it is extremely difficult for specialists to get a handle on this idea at their first gathering. Acknowledgment is the strategy for arranging images. In any case, preparing natural images straightforwardly to image acknowledgment does not create ideal execution. It is advisable that, to get an image contrast, preprocess the image. To further get quality image highlights, diverse image handling procedures are utilized. The information image is preprocessed. The element signals are removed and utilized within the arrangement of the information. The neural organization at that point receives the information of the image and afterward arranges it.

The neural organization's component extractions include a few layers of convolutional and pooled sets. The convolution layer handles the image and rearranges it utilizing the activity of convolution. It is likewise viewed as a work of computerized calculations. The contiguous pixels into one single pixel were consolidated by utilizing pooling layer which eventually lessens the image measurement. Meanwhile ConvNet is connected with two dimensional images, as the conduct of the convolution and pooling layers are easier to be understood in a 2D plane. This is an essential contrast among other neural organizations and ConvNets.

ConvNet suggests the union of the grouping organization and extraction organization. The technique for readiness is utilized for trial computations of the loads of the two layers. The pooling layer has multiple layers of convolution followed by pooling arranged in a stack. The grouping network ordinarily utilizes a normal diverse neural organization for order.

### 5.2. Convolution Layer

The convolution layer is utilized to makes new images which is called highlight maps. The capacity map stresses the surprising parts of the primary image. As opposed to the information layer, the convolution layer is fairly extraordinary by the way it measures input which does not use connection loads and a weighted sum; then again, this layer utilizes filters that change images. These are called spatial channels. The way toward offering contribution to the image is from the convolution channels which give the capacity map. 2D networks are channels in the convolution layers. They are typically accessible as 5 × 5 or 3 × 3 networks. Most recent applications even utilize 1 × 1 channels. The convolution, as it stays on the two-dimensional plane, tends to be an extreme cycle to express in text. Be that as it may, the understanding and computation measures are simpler than they appear. The interaction of convolution is the expansion of the climate items situated in the same places of the two networks.

The qualities accessible in the basics of this element map, identical to the essential convolution activity, rely upon the image framework coordinates with the convolution channel, which is reused without change in measure until the component guide of the given channel is produced.

Toward the end, the element map is made when the convolution layer works the convolution channels on the info image. The skilled convolution channels choose the properties that are extricated in the convolution layer. Thus, contingent upon the convolution channel applied, the properties determined by the convolution layer changes. The component map that convolution channel creates is taken care of with the assistance of the initiation work before the yield layer is produced. The enactment highlight of the layer of convolution resembles that of the standard neural network. Albeit the most extreme current applications utilize the ReLU highlights, alongside the sigmoid capacity and the tanh are also included. The generally utilized moving normal channel in the computerized signal preparing area might be a predominant type of convolution channel for appraisal.

### 5.3. Pooling Layer

The measurements of the image are reduced by the layer of pooling as it presents a combination of adjoining pixels into one delegate worth of a specific image area. The technique of pooling is a standard technique as compared to other diverse image preparing frameworks. To choose the pooling pixel from the image and set the representative worth, neighboring pixels are selected from the framework, and the number of pixels consolidated unique in relation to the issue.

Regularly, an agent esteem is drawn regardless of the line or mean of the assigned pixels. At that point it could be a 2D cycle, the pooling layer measure is shockingly simple. In a numerical strategy, the pooling strategy can be suggested as a means of measure of convolution. The fluctuation from convolution layer is that the convolution channel is fixed, so the spaces of convolution are not covered.

The suggested model given in the resulting area will rely upon this. The layer of pooling makes up for unpredictable and shifted antiquities somewhat. For example, the pooling layer may build a feline's prominence, which might be top centered inside the image. As the image size is getting diminished by the pooling stage, it is exceptionally important to improve the computational overload and deal with overfitting.

### 5.4. Fully Connected Layer

Fully Connected Layers are considered once the Pooling and Convolution layers are added. In a completely connected layer, we connect neurons to all the arrangement of the past layer. Simple lattice improvement is completed along with a predisposition balance. To decrease the number of limitations for managing overfitting issue, a dropout work is utilized inside the completely associated layer. The overfitting will be there when at least two neurons in completely connected layers often recognize similar qualities; neurons are said to have created cotransformation or codependence on one another.

In such a circumstance, a dropout work is applied to diminish this intricacy by overlooking an inconclusive gathering of neurons at the hour of the preparation stage.

One of the weaknesses of utilizing the dropout work is that to accomplish the ideal union stage, more emphasis is required. At last, to recognize the numerals accurately, a Sigmoid classifier is utilized. Loads need to change deliberately on account of beginning haphazardness in loads, and the CNN model should be executed once more. We consider the closeness to the expectations by the unmitigated cross-entropy work and it is a somewhat micro methodology for estimating discrepancies and valid errors.

**Figure 2:**
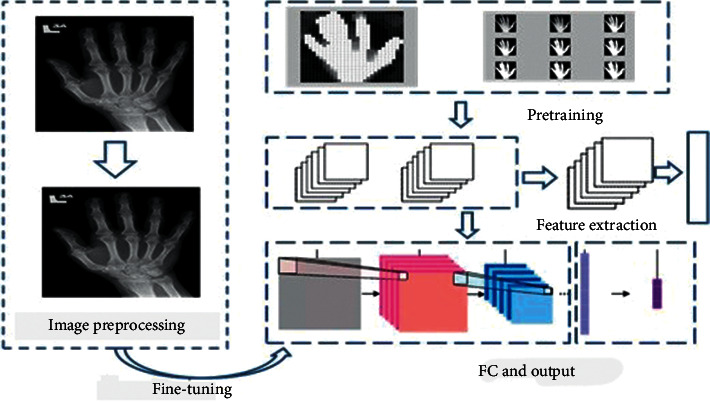
Framework for automatic classification of hand X-ray using convolutional neural network.

Figure 2 shows the framework of automatic classification of hand X-ray.

### 5.5. Classification of Images and Modeling

The model for the picture order dependent on a neural organization of profound convolution is as follows:

1. Input: input is a bunch of *n* pictures, where *n* is any large positive number; one of the *k* characterization labels is each picture mark.
2. Learning: this stage is normally called a classifier for preparing or a learning model. The assignment of this stage is to realize what each class resembles.
3. Assessment: the classifier assigns marks to pictures that have not been seen to gauge the exactness of the classifiers. We liken the marks the classifier predicts with the picture's real names. There is no doubt that the characterization marks the classifier predicts are viable with the genuine picture grouping names.

## 6. Understanding Feature Learning

When a piece for a specific component is characterized, the element is extricated by convolution tasks. For CNN, the quantity of highlights and the idea of a particular component are not characterized ahead of time. In this way, by means of the convolution interaction, no capacity is determined. In forward proliferation, Convolution surmises a capacity and back-engendering endeavors to sequentially address the presumption.

Various portions after complete readiness are balanced out utilizing a mistake minimization strategy. Convolution attempts to positively avoid every attribute that cannot be chosen until after this arrangement has been completed. In the proposed model, obscure hand X-beam qualities are divided by convolution layers, with 1 layer having 20 portions. The essential presence of each capacity is seen following the preparation.

**Figure 3:**
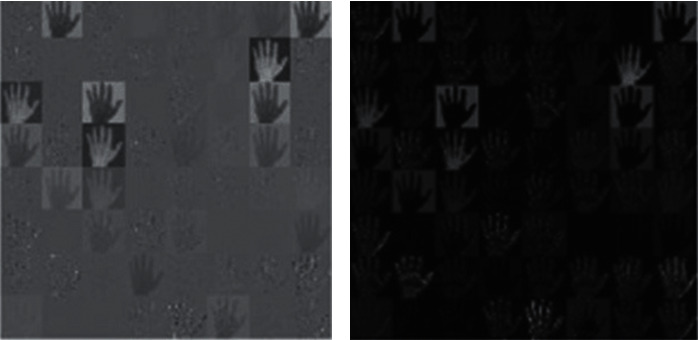
Output of different layer. (a) Convolution layer. (b) Pool layer.

Convolution layer is shown in subpart (a) of Figure 3 and pool layer yield in subpart (b) of the same figure.

## 7. Experimental Results

**Figure 4:**
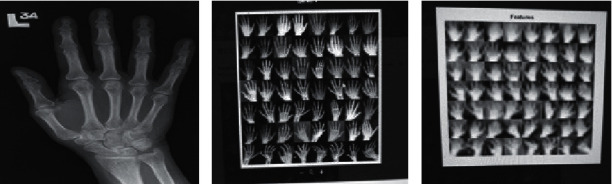
Training of hand X-ray thorough system. (a) Original hand X-ray image. (b) Input images for training. (c) Featured images during training.

**Figure 5:**
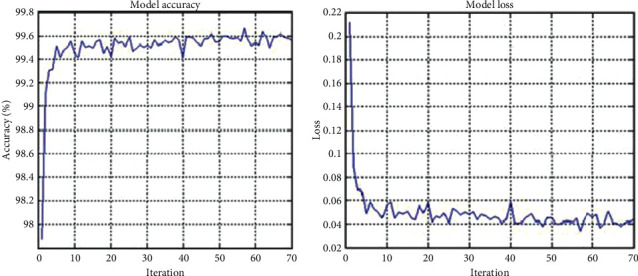
Model performance. (a) Model accuracy. (b) Model loss.

For executing the CNN model, a MATLAB® programming cloud service was utilized. The setup was established with a dataset of about 218 radiographical images of the handout of which 61 were standard and 120 had RA. At that point, 72 radiographs, comprising 32 standard and 40 RA tests, were used to test the system.  Figure 4(a) shows the original pictures, Figure 4(b) shows the input pictures, and Figure 4(c) shows the featured pictures. For our proposed model, plots of exactness and misfortune over numerous cycles are presented in Figures 5(a) and 5(b).

It is evident that preparation exactness (blue line) and approval precision (dark line) approach each other during the preparation cycle. The loss subplot likewise shows that both the deficiency of preparing and the deficiency of approval are low and decline during the interaction. The proposed model reports improvement in accuracy as compared with CNN models previously executed. The popular MATLAB@2020 Design Deep Learning App is utilized for doing this.

Our model is compared with other standard classifiers such as SVM and ANN and the proposed model presents a better classification accuracy.

## 8. Methods Employed for Comparison

The determination of the classifier is the critical element that influences the programmed exactness of discovery of the hand X-beam dependent on highlight extraction by a similar strategy for misfortune work conducted by the machine. The result of a few classifiers on exactness of grouping is discussed in this article.  Table 3 gives the impact on characterization exactness of different standard classifiers. These classifiers are linear SVM and ANN.

The presented results show that the accuracy of the CNN classifier in the training phase and test set is higher than that of different classifiers which shows that a noteworthy accuracy is obtained through the presented method, i.e., by CNN. First and foremost, it requires a tremendous extra room, sets aside a ton of effort to gauge, and uses a great deal of computational force. Because of this, all the put away preparing pictures needed to be contrasted. Here in this article, the more focus is given on estimating execution much effectively than showing proficiency practically speaking. As a general rule, the CNN has certain limitations: while the training of the model takes a great deal of time, the arrangement of new test information is fast once the training of the model is complete.

**Figure 6:**
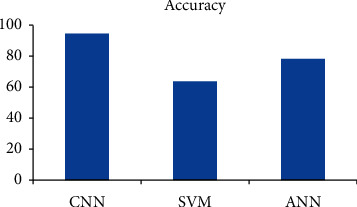
Diagnostic accuracy comparison for the same set of images for all the graphs.

**Figure 7:**
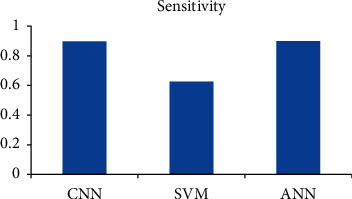
Diagnostic sensitivity comparison.

**Figure 8:**
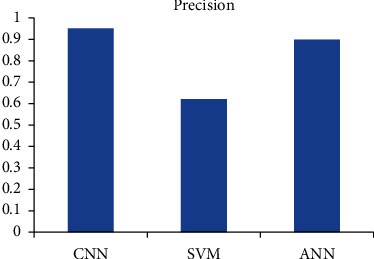
Diagnostic precision comparison.

Figure 6 shows the diagnostic accuracy graph for the algorithms.  Figure 7 shows the sensitivity and Figure 8 shows the precision.

### 8.1. Comparison with CNN Lenet

**Figure 9:**
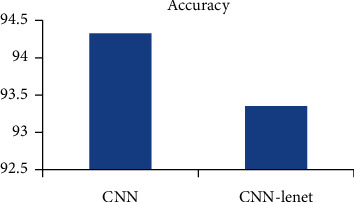
Accuracy: CNN and Lenet.

The CNN Lenet and CNN are compared to find out the accuracy. By training both networks it is found that CNN is better than Lenet which is shown in Figure 9.

## 9. Conclusions

A decision of RA is made by assessing the lab reports and imaging especially hand X-beam. Radiography is an effectively available imaging technique; the expert in surveying RA discoveries utilizing radiography is significant and simultaneously it is a ling tedious interaction. In the early treatment of RA finding permanent deformities found in RA can be forestalled. So mechanized technique for distinguishing RA becomes useful. In this examination we prepared and assessed CNN and CNN Lenet model. Likewise, the near examination is finished with SVM and artificial neural organization GRNN. We propose a framework that distinguishes RA without broad preprocessing of radiographic hand X-beam pictures.

The proposed model zeroed in on the programmed characterization of hand X-beam for rheumatoid arthritis patients with the information picture dataset of 290 pictures. The proposed model presents a model accuracy of approximately 95%. This model is utilized to gain proficiency with the hand X-beam including consequently. The programmed recognition of the typical and unusual condition is tried effectively and furthermore confirmed with the specialist. The proposed model is contrasted and compared with diverse popular CNN techniques and reports improved accuracy. It is additionally contrasted and various classifiers. The proposed strategy includes improved exactness and accuracy.

In future we desire to increase the dataset size with the prediction of phases of RA even before the transitional and serious stage.

## Figures and Tables

**Table 1:** Description of dataset.

Total cases	290
Normal cases	130
RA cases	160

**Table 2:** Number of images for training and testing.

Cases	Training	Test
Normal cases	98	32
RA cases	120	40

**Table 3:** Comparison of popular methods with the proposed method.

Method	Author/s	Year	No. of samples	Precision	Specificity	Sensitivity	Accuracy (%)
CNN	Stoel [21]	2019	135	0.75	0.78	0.68	73.33
CNN lenet	Betancourt-Hernández et al. [8]	2018	92	—	—	—	93.00
Proposed method	—	—	290	0.95	0.92	0.96	94.64

## Data Availability

Data are available on request to the corresponding author.
