# Corrigendum to “An Efficient CNN for Hand X-Ray Classification of Rheumatoid Arthritis”

**DOI:** 10.1155/2021/9808309

**Published:** 2021-10-11

**Authors:** Gitanjali S. Mate, Abdul K. Kureshi, Bhupesh Kumar Singh

**Affiliations:** ^1^Department of Electronics and Telecommunication, JSPM's Rajarshi Shahu College of Engineering, Pune 411033, India; ^2^Department of Electronics, Maulana Mukhtar Ahmad Nadvi Technical Campus, Malegaon 423203, India; ^3^Arba Minch Institute of Technology, Arba Minch University, Arba Minch, Ethiopia

In the article titled “An Efficient CNN for Hand X-Ray Classification of Rheumatoid Arthritis” [1], reference 8 is listed incorrectly and should be corrected as [2].
